# Engineering zinc oxide hybrid selenium nanoparticles for synergetic anti-tuberculosis treatment by combining Mycobacterium tuberculosis killings and host cell immunological inhibition

**DOI:** 10.3389/fcimb.2022.1074533

**Published:** 2023-01-26

**Authors:** Wensen Lin, Shuhao Fan, Kangsheng Liao, Yifan Huang, Yanguang Cong, Junai Zhang, Hua Jin, Yi Zhao, Yongdui Ruan, Hongmei Lu, Fen Yang, Changxian Wu, Daina Zhao, Zhendong Fu, Biying Zheng, Jun-Fa Xu, Jiang Pi

**Affiliations:** ^1^ Guangdong Provincial Key Laboratory of Medical Molecular Diagnostics, The First Dongguan Affiliated Hospital, Guangdong Medical University, Dongguan, China; ^2^ Institute of Laboratory Medicine, School of Medical Technology, Guangdong Medical University, Dongguan, China; ^3^ Songshan Lake Materials Laboratory, Dongguan, China

**Keywords:** tuberculosis, zinc oxide-selenium nanoparticles, macrophages, anti-TB agents, killing, immunological inhibition

## Abstract

**Introduction:**

As a deadly disease induced by Mycobacterium tuberculosis (Mtb), tuberculosis remains one of the top killers among infectious diseases. The low intracellular Mtb killing efficiency of current antibiotics introduced the long duration anti-TB therapy in clinic with strong side effects and increased drug-resistant mutants. Therefore, the exploration of novel anti-TB agents with potent anti-TB efficiency becomes one of the most urgent issues for TB therapies.

**Methods:**

Here, we firstly introduced a novel method for the preparation of zinc oxide-selenium nanoparticles (ZnO-Se NPs) by the hybridization of zinc oxide and selenium to combine the anti-TB activities of zinc oxide nanoparticles and selenium nanoparticles. We characterized the ZnO-Se NPs by dynamic laser light scattering and transmission electron microscopy, and then tested the inhibition effects of ZnO-Se NPs on extracellular Mtb by colony-forming units (CFU) counting, bacterial ATP analysis, bacterial membrane potential analysis and scanning electron microscopy imaging. We also analyzed the effects of ZnO-Se NPs on the ROS production, mitochondrial membrane potential, apoptosis, autophagy, polarization and PI3K/Akt/mTOR signaling pathway of Mtb infected THP-1 macrophages. At last, we also tested the effects of ZnO-Se NPs on intracellular Mtb in THP-1 cells by colony-forming units (CFU) counting.

**Results:**

The obtained spherical core-shell ZnO-Se NPs with average diameters of 90 nm showed strong killing effects against extracellular Mtb, including BCG and the virulent H37Rv, by disrupting the ATP production, increasing the intracellular ROS level and destroying the membrane structures. More importantly, ZnO-Se NPs could also inhibit intracellular Mtb growth by promoting M1 polarization to increase the production of antiseptic nitric oxide and also promote apoptosis and autophagy of Mtb infected macrophages by increasing the intracellular ROS, disrupting mitochondria membrane potential and inhibiting PI3K/Akt/mTOR signaling pathway.

**Discussion:**

These ZnO-Se NPs with synergetic anti-TB efficiency by combining the Mtb killing effects and host cell immunological inhibition effects were expected to serve as novel anti-TB agents for the development of more effective anti-TB strategy.

## Introduction

Tuberculosis, induced by *Mycobacterium tuberculosis* (Mtb), remains one of the top killers among infectious diseases up to now (World Health Organization, 2021). The emergence of some potent anti-TB antibiotics, such as rifampicin, ethambutol and isoniazide, have dramatically saved millions of lives worldwide, however, also brings some new urgent issues that significantly threatening the public health. The low intracellular Mtb killing efficiency of current antibiotics introduced the long duration anti-TB therapy in clinic, which not only result in the widely reported side effects, but also lead to the emergence of more dangerous drug-resistant mutants (Parida et al., 2015; Petros et al., 2016). Therefore, the exploration of novel anti-TB agents with potent anti-TB efficiency becomes one of the most urgent issues for TB therapies.

Taking the advantages of the ability for engineering of functional systems at the molecular scale, nanotechnology can be applied for the use of matter on an atomic, molecular, and supramolecular scale for different purposes, including industrial and medical (Surendiran et al., 2009). Nanomaterials-based nanomedicines are now attracting increasing attentions worldwide due to their improved efficacy, bioavailability, dose-response, targeting ability, personalization, and safety compared to conventional medicines (Choi and Han, 2018; Foulkes et al., 2020). In recant decades, lots of functional nanomaterials have been developed into clinical diseases treatment (Min et al., 2015; Bobo et al., 2016), which therefore provide new possibilities for the development of novel anti-TB strategies.

Unlike antibiotics, the use of nanomaterials for bacterial infection treatment wouldn’t introduce the drug resistance issues (Gupta et al., 2019). In recent years, some promising nanomaterials have been highlighted for their potential uses in TB treatment, based on their direct Mtb inhibition/killing effects, drug delivery ability or immunological regulation effects (Xu et al., 2018). Zinc oxide nanoparticles (ZnO NPs), with large surface area relative to their size and high catalytic activity, are widely reported in biomedical fields due to their potent anticancer and antimicrobial activities (Jiang et al., 2018). Very interestingly, the ZnO NPs with low cytotoxicity are recently found to show strong inhibition effects on Mtb growth (Heidary et al., 2019; Behzad et al., 2022), which therefore are expected to be engineered into anti-TB agents. However, the anti-TB efficiency of ZnO NPs remains to be further improved.

Based on the essential element selenium, some functional selenium nanoparticles (Se NPs) have been developed for anticancer and antibacterial treatment in recent decades (Ferro et al., 2021; Chen et al., 2022). Se NPs have also been shown to kill and inhibit Mtb directly, indicating their anti-TB application potentials (Estevez et al., 2020). And in our recent work, we have clearly demonstrated that Se NPs could serve as potent anti-TB agents to kill both extracellular and intracellular Mtb with host cell immunity activation responses (Pi et al., 2020). However, the strong cytotoxicity of Se NPs to the host cells seriously restricted their further anti-TB applications.

Here, combining our nanotechnology (Huang et al., 2021a; Huang et al., 2021b) and anti-TB expertise (Pi et al., 2020; Luo et al., 2021; Pi et al., 2022), we firstly introduced a novel method for preparation of zinc oxide-selenium nanoparticles (ZnO-Se NPs) to combine the anti-TB activities of zinc oxide nanoparticles and selenium nanoparticles. By investigating their potential anti-TB effects and mechanisms, our results indicated ZnO-Se NPs as a kind of novel anti-TB agents that might benefit the future anti-TB strategy development.

## Materials and methods

### Materials

Sodium selenite and ascorbic acid were purchased from Sinopharm Chemical Reagent (China). 7H9 medium, 7H11 medium and OADC were ordered from BD (USA). rhodamine123, zinc acetate, sodium hydroxide, DIOC2(3)(3,3’- Diethyloxacarbocyanine Iodide) were obtained from Macklin (Shanghai, China). Dialysis bags (MWCO: 8000-14,000), 1640 medium, protein assay kit, EDTA-trypsin, SDS, 4% paraformaldehyde and penicillin/streptomycin were purchased from Solaibao (Beijing, China). Protein lysis buffer, nitrite assay kit, GAPDH antibody and anti-rabbit IgG were obtained from Beyotime Institute of Biotechnology (Shanghai,China). APC anti-human CD80 antibody, PE anti-human CD163 antibody, APC anti-human TNF-α antibody, FITC anti-human IFN-γ antibody, PE anti-human IL-10 antibody were purchased from Biolegend(USA). Fix/perm kit was purchased from BD (USA). PAA[Poly(acrylic acid)], Lyso-Tracker Red kit and DAPI were ordered from Sigma-Aldrich (USA). LC3B antibody, p-PI3K antibody and Alexa Fluor^®^ 555 Goat Anti-Rabbit IgG Ab were provided by Abcam (UK). p-AKT antibody and p-mTOR antibody were obtained from Cell Signaling Technology (USA). Other regents were listed as follows: FBS (BI,USA), Bac Titer-Glo^®^ reagent (Promega, USA), Annexin V-FITC/PI apoptosis detection kit (Multisciences, China), Propidium Iodide (PI; Shanghai YuanYe Biotechnology, China), 2,7-dichlorodihydrofluorescein diacetate (DCFDA; Shanghai DiBo Biotechnology, China).

### Preparation and characterization of ZnO-Se NPs

100 mg polyacrylic acid was dissolved in 40 ml water under stirring, then 0.5 ml sodium selenite solution (50 mM) was added, followed by the addition of 2 ml of ascorbic acid solution (100 mM) for 1 hour stirring. After that, 0.11 g of zinc acetate in 10 ml water was added, followed by the addition of 1.1 ml of NaOH (2M) for another 1 hour stirring. The obtained ZnO-Se NPs were purified by dialysis against distilled water to remove the excess reactants and other by-products. The as-prepared ZnO-Se NPs were characterized by dynamic laser light scattering (DLS, Horiba, Japan) and transmission electron microscopy (TEM, JEL, Japan).

### Bacteria and cell culture

BCG and H37Rv were obtained from ATCC (USA). And both BCG and H37Rv were all cultured in 7H9 medium with 10% OADC enrichment. THP-1 cells was obtained from ATCC (USA) and cultured with 1640 medium supplemented with 10% FBS in a humidified atmosphere of 5% CO at 37°C. All experiments about H37Rv are performed in BSL-3 labs.

### Effects of ZnO-Se NPs on the growth of extracellualr BCG

4×10^5^ colony-forming units (CFU) of BCG suspension in 7H9 medium was added into a 2 mL tube with different concentration of ZnO-Se NPs in 7H9 medium at 37 °C incubator for 72 h incubation. After that, the suspension from different groups were diluted by 7H9 medium, and then plated on Middlebrook 7H11 plates. CFU counts of BCG on plates were counted after 3-4 weeks in the incubator at 37 °C.

### Preparation of SEM samples

4×10^5^ colony-forming units (CFU) of BCG suspension in 7H9 medium was added into a 2 mL tube with or without ZnO-Se NPs in 7H9 medium. Then, the suspension of BCG were dropped onto small glass slides for 2h following the overnight fixation of BCG or H37Rv by 2.5% glutaraldehyde and 2% paraformaldehyde for 24 h. After that, the fixed BCG were washed by PBS and pure water, and further treated with 50%, 70%, 85%, 95%, 100% ethanol and HMDS (Hexamethyldisilazane), respectively. Then, the samples were gold sputter-coated and observed by a Field Emission SEM (JEOL, Japan).

### Flow cytometry analysis of BCG death and ROS production

BCG suspension (1×10^6^ CFU) in 7H9 medium was added into a 2 mL tube with or without ZnO-Se NPs in the incubator. Then, after 3h incubation, the bacteria were collected, washed with PBS and stained with 2’,7’- Dichlorodihydro fluorescein Diacetate for 30 min, followed by flow cytometry (BD, USA) analysis. And after 72 h incubation, the bacteria were collected, washed with PBS and stained with PI for 30 min, followed by flow cytometry (BD, USA) analysis.

### Bacterial ATP analysis

For extracellular bacterial ATP analysis, 200 μL 2×10^6^ colony-forming units (CFU) of BCG suspension were grown in white opaque bottom 96-well plates with different concentration of ZnO-Se NPs. After 24 h incubation, 100 μL of culture was taken from each well and mixed with 100 μL of Bac Titer-Glo^®^ reagent. Then, the assay was performed according to the manufacturer’s instructions. Luminescence was measured on the Gen5 Microplate reader (Biotek, USA) at an integration time of 500 milliseconds.

### Bacterial membrane analysis

For Bacterial membrane potential analysis, 2×10^6^ CFU of BCG suspension was treated with different concentration of ZnO-Se NPs in 7H9 medium at 37°C incubator for 72h incubation. Then, the collected bacteria were centrifugated, washed with PBS and stained with DIOC2(3) for 30 min at room temperature, which was further washed with PBS and analyzed by flow cytometry (BD, USA).

### Effects of ZnO-Se NPs on the growth of intracellualr BCG in THP-1 cells

THP-1 cells were seeded at a density of 4×10^5^ into 12 well plates with 100 nM PMA stimulation for 24 h incubation. Then, the cells were infected with BCG using a multiplicity of infection (MOI) of 1 for 24 h. After washed with PBS to clean the Mtb outside the cells, different concentrations of ZnO-Se NPs were added into the cells for 72 h incubation. After that, 0.03% SDS solution was used to lyse the cells for 15 min. The cell lysis were plated on Middlebrook 7H11 plates after dilution with 7H9 medium, and the CFU on plates were counted after 3-4 weeks in a 37°C incubator.

### Cell polarization analysis

THP-1 cells were seeded at a density of 1×10^6^ into 12 well plates with 100 nM PMA stimulation for 24 h incubation, and then infected with BCG using a MOI of 1 for 24 h. After washed with PBS to clean the BCG outside the cells, different concentrations of ZnO-Se NPs were added into the cells for 24 h incubation. Cells were then collected, washed with PBS, stained with APC anti-human CD80 antibody and PE anti-human CD163 antibody, and then analyzed by flow cytometry (BD, USA). For intracellular cytokines analysis, THP-1 cells were seeded at a density of 1×10^6^ into 12 well plates with 100 nM PMA stimulation for 24 h incubation, and then infected with BCG using a MOI of 1 for 24 h. After washed with PBS to clean the BCG outside the cells, different concentrations of ZnO-Se NPs were added into the cells for 24 h incubation. Cells were then collected, washed with PBS, and then treated with fix/perm kit following the instructions. Then, cells were incubated with APC anti-human TNF-α antibody, FITC anti-human IFN-γ antibody and PE anti-human IL-10 antibody, and then analyzed by flow cytometry (BD, USA).

### Cell apoptosis analysis

THP-1 cells were seeded at a density of 1×10^6^ into 12 well plates with 100 nM PMA stimulation for 24 h incubation, and then infected with BCG using a MOI of 1 for 24 h. After washed with PBS to clean the BCG outside the cells, different concentrations of ZnO-Se NPs were added into the cells for 24 h incubation. Cells were then collected, washed with PBS, stained with APC Annexin V/7-AAD apoptosis kit following the manufacturer’s protocol, and then analyzed by flow cytometry (BD, USA).

### Immunofluorescence microscopy analysis

THP-1 cells were seeded at a density of 2×10^5^ into confocal dishes with 100 nM PMA stimulation for 24 h incubation, and then infected with BCG using a MOI of 1 for 24 h. After washed with PBS to clean the BCG outside the cells, ZnO-Se NPs were added into the cells for 24 h incubation. The cells were washed with PBS, and then were fixed by 4% paraformaldehyde for 10 min, permeabilized with 0.2% Triton X-100/PBS for 10 min, and pre-blocked in 5% BSA/PBS overnight. The cells were then incubated with anti-rabbit LC3B (Microtubule-associated protein 1A/1B-light chain 3B) antibody that was diluted at 1/100 in blocking solution for 24 h, washed three times with PBS, and incubated with Alexa fluor 488-conjugated anti-rabbit IgG Ab (1/200 in blocking solution) for 1 h. After washed with PBS, the cells were further incubated with 10 μg/mL hochest33342 for 20 min, and then used for confocal microscopy (Leica, German) analysis after PBS wash.

### Western-Blot analysis

THP-1 cells were seeded at a density of 1×10^6^ into 12 well plates with 100 nM PMA stimulation for 24 h incubation, and then infected with BCG using a MOI of 1 for 24 h. After washed with PBS to clean the BCG outside the cells, different concentrations of ZnO-Se NPs were added into the cells for 24 h incubation. THP-1 cells were incubated with lysis buffer containing protease inhibitors to obtain total cellular proteins. The protein concentration in the lysate was measured using the Bio-Rad protein assay kit. Proteins were then denatured by boiling at 100°C for 10 min in sample buffer. The samples were then separated by electrophoresis on 10% SDS-polyvinylamide minigels, after which, they were transferred to polyvinylidene difluoride membranes. The transferred membranes were blocked with 5% skim milk in TBST solution for 1 h, and then washed three times with Tris-Buffered Saline and Tween 20 (TBST) (5 min each time). After incubated with primary antibodies, including LC3B antibody, phosphorylation-PI3K antibody, phosphorylation-AKT antibody, phosphorylation-mTOR antibody or GAPDH antibody overnight at 4°C, the membranes were washed three times with TBST, and incubated with secondary antibodies (Cell Signaling, USA) at room temperature for 1 h followed by wash with TBST (Tris Buffered Saline Tween) and ECL (Electrochemiluminescence) detection.

### Intracellular ROS (Reactive Oxygen Species) and mitochondrial membrane potential measurements of THP-1 cells

THP-1 cells were seeded at a density of 1×10^6^ into 12 well plates with 100 nM PMA stimulation for 24 h incubation, and then infected with BCG using a MOI of 1 for 24 h. After washed with PBS to clean the BCG outside the cells, cells were treated with ZnO-Se NPs at various period of time. After incubated with ROS detection reagent (Sigma) for 30 min, cells were analyzed by a flow cytometry (BD, USA). And for mitochondiral membrane potential analysis, different concentrations of ZnO-Se NPs were added into the BCG infected THP-1 cells for 24 h incubation. After that, cells were then stained with rhodamine123 for 30 min, followed by flow cytometry analysis (BD, USA).

### Effects of ZnO-Se NPs on the growth of extracellular H37Rv and intracellualr H37Rv in THP-1 cells

4×10^5^ colony-forming units (CFU) of H37Rv suspension in 7H9 medium was added into a 2 mL tube with different concentration of ZnO-Se NPs in 7H9 medium. The tubes were then slowly rotated in the incubator at 37 °C for 72 h. After that, the suspension from different groups were diluted by 7H9 medium, and then plated on Middlebrook 7H11 plates. CFU counts on plates were counted after 3-4 weeks in the incubator at 37 °C. THP-1 cells were seeded at a density of 4×10 ^5^ into 12 well plates with 100 nM PMA stimulation for 24 h incubation. Then, the cells were infected with H37Rv using a multiplicity of infection (MOI) of 1 for 4 h. After washed with PBS to clean the Mtb outside the cells, different concentrations of ZnO-Se NPs were added into the cells for 72 h incubation. After that, 0.03% SDS solution was used to lyse the cells for 15 min. The cell lysis were plated on Middlebrook 7H11 plates after dilution with 7H9 medium, and the CFU on plates were counted after 3-4 weeks in a 37°C incubator.

### Statistical analysis

All experiments were repeated three times and results were expressed as mean ± standard deviation (SD). All statistics were analyzed using GraphPad Prism 8 software (GraphPad, USA), which were applied using nonparametric t test between two groups or one-way ANOVA test when the experiment groups were more than two. P-value < 0.05 was regarded as statistically significant.

## Results and discussion

### Preparation and characterization of ZnO-Se NPs

**Figure 1:**
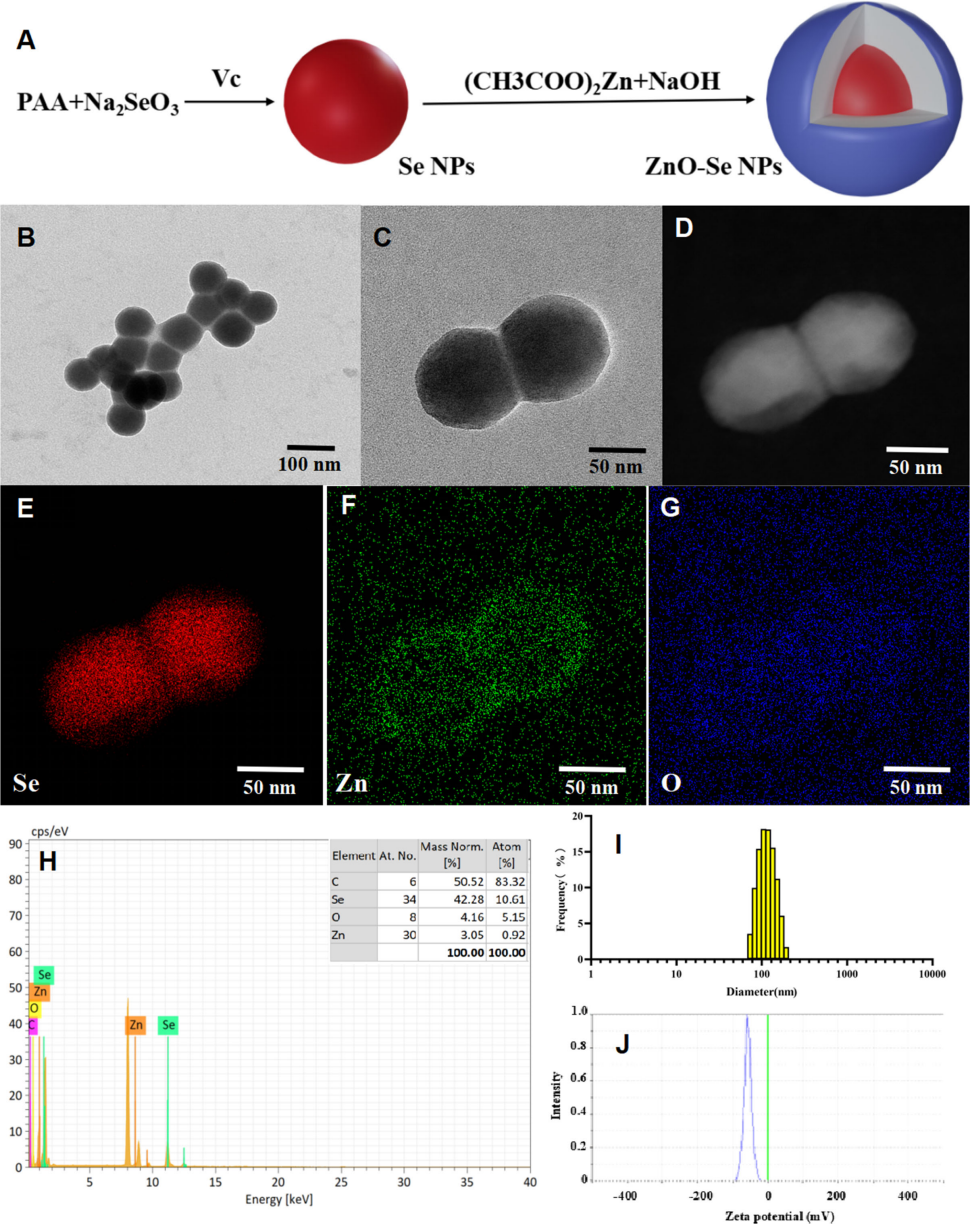
Preparation and characterization of ZnO-Se NPs. **(A)** Schemes for the preparation of ZnO-Se NPs. **(B, C)** Typical TEM images of ZnO-Se NPs. **(D)** Typical TEM-EDS dark field image of ZnO-Se NPs. **(E)** Typical TEM-EDS selenium (Se) mapping analysis of ZnO-Se NPs. **(F)** Typical TEM-EDS znic (Zn) mapping analysis of ZnO-Se NPs. **(G)** Typical TEM-EDS oxygen (O) mapping analysis of ZnO-Se NPs. **(H)** TEM-EDS elemental analysis of ZnO-Se NPs. **(I)** Size analysis of ZnO-Se NPs by DLS. **(J)** Zeta potential analysis of ZnO-Se NPs by DLS.

Based on their attractive biological activities, ZnO NPs and Se NPs have both showed potential anti-TB application in recent years (Heidary et al., 2019; Estevez et al., 2020; Pi et al., 2020; Behzad et al., 2022). Thus, the combination of ZnO NPs and Se NPs are expected to show synergistic anti-TB effects in theory. But the simple mixtures of ZnO NPs and Se NPs might also introduce some unexpected issues, such as poor stability and low biocompability. Alloy nanoparticles have been widely reported to show enhanced chemical, physical or biological activities beyond the the simple mixture of different kind of mono nanoparticles (Ibañez and Zamborini, 2012; Huynh et al., 2020). To achieve novel anti-TB candidates by nanotechnology, we combined the antibacterial advantages of ZnO NPs and Se NPs together to engineer a kind of novel alloy nanoparticles, naming ZnO-Se NPs as shown in **Figure 1A**. Here, polyacrylic acid (PAA) was used as stabilizers to form Se NPs after the redox reaction between sodium selenite and ascorbic acid. After that, sodium hydroxide turned zinc acetate into zinc hydroxide, which further dehydrate to form zinc oxide and cover onto the surface of Se NPs core to achieve ZnO-Se NPs (**Figure 1A**). As shown in **Figures 1B, C**, TEM imaging of the obtained ZnO-Se NPs showed spherical morphology of ZnO-Se NPs with an average diameter of 90 nm. Using elemental mapping analysis by TEM-EDS, we could found the core-shell structure of ZnO-Se NPs with selenium nanoparticle core inside and surface coating of ZnO outside the selenium nanoparticles (**Figures 1D–G**). The statistical element analysis by TEM-EDS also indicated the zinc element and selenium element in the obtained nanoparticles (**Figure 1H**), indicating the successful formation of zinc oxide and selenium hybrid ZnO-Se NPs.

DLS analysis further indicated that the average hydrous diameter of ZnO-Se NPs ranged from 80 nm to 400 nm with an average diameter of 130 nm (**Figure 1I**). These results indicated the formation of a thick polymeric and hydrous coating (~20 nm in radius, formed by polyacrylic acid) around the ZnO-Se core that could not be clearly observed by TEM imaging, which provided an ideal warehouse for the encapsulation of hydrophobic guest molecules for further drug delivery. Additionally, zeta potential was also analyzed by DLS, which indicated that the average zeta potential of the obtained ZnO-Se NPs was -60 mV (**Figure 1J**), indicating their strong surface charges that are helpful to prevent the aggregation of nanoparticles. To further evaluate the stability of ZnO-Se NPs, we determined the changes of size distribution and zeta potential of ZnO-Se NPs, which indicated no significant size changes during 10 days storage (**Supplementary Material Figure 1**). Moreover, the average zeta potential of ZnO-Se NPs kept at more than -40 mV during the 10 days storage, which also suggested their strong stability (**Supplementary Material Figure 1**).

### Direct extracellular Mtb inhibition/killing effects of ZnO-Se NPs

Our previous work has demonstrated the strong Mtb inhibition effects of Se NPs (Pi et al., 2020), which indicated the promising potentials of ZnO-Se NPs for anti-TB strategy development. To explore whether ZnO NPs could relieve the cytotoxicity of Se NPs against host cells for more effective anti-TB treatment, we determined the viability of ZnO-Se NPs against THP-1 cells. 10 μg/mL ZnO-Se NPs didn’t induce significant cell proliferation inhibition in THP-1 cells and RAW264.7 cells after 24 h treatment (**Supplementary Material Figure 2**), which indicated that 10 μg/mL ZnO-Se NPs could be used as a nontoxic dosage for the following anti-TB treatment. Interestingly, the nontoxic dosage of ZnO-Se NPs (below 10 μg/mL) even showed weak ability to enhance the proliferation of THP-1 macrophages (**Supplementary Material Figure 2**), which might be attributed to the selenium and zinc supplementation to support THP-1 macrophage growth.

**Figure 2:**
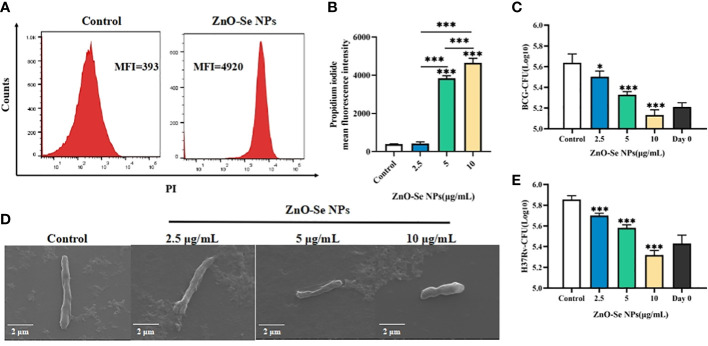
Killing/inhibition effects of ZnO-Se NPs against Mtb. **(A)** Typical flow cytometry analysis of PI signals for BCG before and after 10 μg/mL ZnO-Se NPs treatment. **(B)** Statistical results of PI signals for BCG before and after ZnO-Se NPs treatment, n=3, ***p<0.001. **(C)** Effects of ZnO-Se NPs on the growth of extracellular BCG, n=3, *p<0.05, ***p<0.001. **(D)** SEM images of BCG before and after ZnO-Se NPs treatment, scale bar: 2 μm. **(E)** Effects of ZnO-Se NPs on the growth of extracellular H37Rv, n=3, ***p<0.001.

To further investigated the anti-TB potentials of ZnO-Se NPs, we investigated the inhibition effects of ZnO-Se NPs on BCG, a kind of live attenuated strain of Mycobacterium bovis that widely used as TB vaccines and Mtb research model. Firstly, we analyzed the death of BCG by the DNA staining of dead cells with PI. The obtained results demonstrated much stronger PI signal in ZnO-Se NPs treated BCG (**Figures 2A, B**), which indicated the strong destruction effects of ZnO-Se NPs to induce Mtb death. The CFU counting results also indicated that ZnO-Se NPs could significantly inhibit BCG growth with non-cytotoxic concentrations against host cells, and 10 μg/mL ZnO-Se NPs treated group even showed lower CFU of BCG than that of Day0 control (**Figure 2C**). And by SEM imaging, the typical bacilli form morphology was observed in control BCG group, however, significantly shrunk morphology of BCG was observed in ZnO-Se NPs treatment group (**Figure 2D**). And in high dosage of ZnO-Se NPs treated group, some BCG were broken into smaller pieces or fragments with wrinkled appearance (**Figure 2D**), which indicated that ZnO-Se NPs could kill Mtb and disrupt their morphology directly. More importantly, we also verified the effects of ZnO-Se NPs on the virulent Mtb-H37Rv, which also showed similar inhibition growth effects on H37Rv (**Figure 2E**), indicating the strong Mtb killing effects of ZnO-Se NPs.

### ZnO-Se NPs kill Mtb by disrupting intracellular ATP production, ROS level and destroying cell membrane integrity

**Figure 3:**
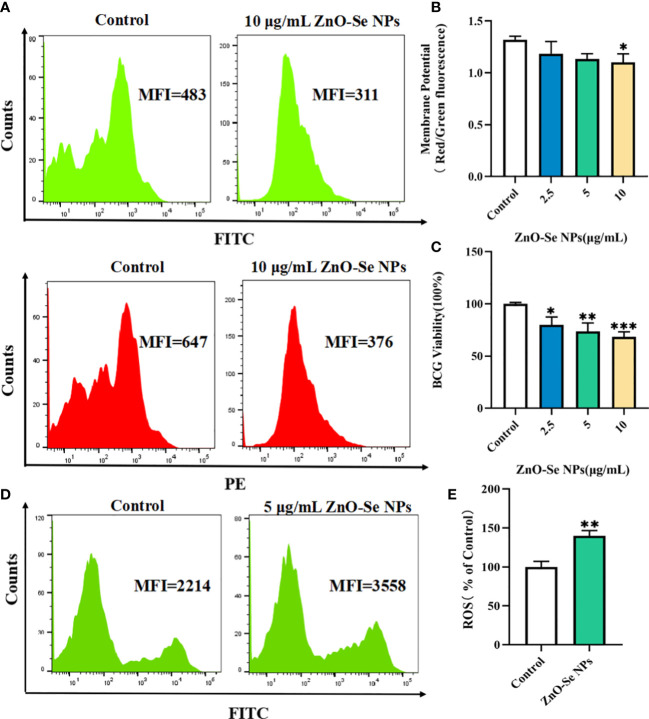
Killing/inhibition mechanisms of ZnO-Se NPs against Mtb. **(A)** Typical flow cytometry analysis of membrane green and red signals for BCG before and after 10 μg/mL ZnO-Se NPs treatment. **(B)** Statistical results of membrane potential for BCG before and after ZnO-Se NPs treatment, n=3, *p<0.05. **(C)** Viability analysis by detecting the ATP production of BCG before and after ZnO-Se NPs treatment, n=3, *p<0.05, **p<0.01, ***p<0.001. **(D)** Typical flow cytometry analysis of ROS levels for BCG before and after 5 μg/mL ZnO-Se NPs treatment. **(E)** Statistical results of ROS level for BCG before and after ZnO-Se NPs treatment, n=3, **p<0.01.

To further explore the underlying mechanisms for the anti-TB activity of ZnO-Se NPs, we investigated the effects of ZnO-Se NPs on the intracellular ATP production, ROS production, and membrane integrity of BCG. Bacterial cell membranes are served as a promising target for developing new antibacterial drugs since some important biomolecules, such as the membrane-based efflux pump systems, play an important role in bacterial pathogenicity and antimicrobial resistance in bacteria (Chitemerere and Mukanganyama, 2014). Thus, we used DIOC2(3) for cell membrane potential analysis. DIOC2(3) can be uptaken by bacteria to show green fluorescence in the bacteria cells. The high membrane potential can induce the self-polymerization of DIOC2(3) to show the fluorescence shift from green (FITC channel) to red (PE channel). Thus, the higher membrane potential of bacteria would show higher red/green fluorescence ratio, and the lower membrane potential would show lower red/green fluorescence ratio. As shown in **Figures 3A, B**, we found that high dosage of ZnO-Se NPs treatment (10 μg/mL) significantly disrupted the membrane integrity of BCG through the membrane potential staining. These results demonstrated that ZnO-Se NPs killed Mtb was closely associated with their ability to disrupt the bacterial membrane structures and functions.

Bacteria use the sugars they produce or obtain to produce cellular energy, naming adenosine triphosphate (ATP), which is important for different kinds of biological functions of bacteria to keep them alive (Hironaka et al., 2013). Due to the important roles of ATP in Mtb, it has also been proved that targeting the ATP production of Mtb could be served as clinical anti-TB strategy (Lakshmanan and Xavier, 2013). By analyzing the intracellular ATP contents using BacTiter-Glo microbial cell viability assay, we also found that ZnO-Se NPs treatment could significantly decrease the cell viability by reducing the ATP production of BCG (**Figure 3C**), which demonstrated that ZnO-Se NPs could also disrupt the ATP production of Mtb to induce Mtb death.

Some agents that modulate antioxidant levels and/or enhance intracellular ROS in bacteria could disturb the cellular oxidative environment and induce cell death, and hence could serve as novel therapeutics (Dharmaraja, 2017; Van Acker and Coenye, 2017). The intracellular ROS of BCG was found to be significantly elevated even after very low dosage of ZnO-Se NPs treatment (**Figures 3D, E**), which suggested that ROS dysfunction was closely related to ZnO-Se NPs killed Mtb. Thus, these results collectively demonstrated that ZnO-Se NPs could kill Mtb directly by reducing intracellular ATP production, increasing intracellular ROS production and disrupting the membrane integrity of BCG.

### ZnO-Se NPs promote polarization of Mtb infected macrophages

Functional skewing of monocyte/macrophage polarization always occurs in physiological conditions (e.g., ontogenesis and infection), which is now considered as a key determinant of disease development and regression (Sica et al., 2015). M1 type macrophages release high levels of pro-inflammatory cytokines and nitric oxide (NO) to exhibit high anti-mycobacterial activity; on the contrary, M2 type macrophages produce inhibitory cytokines that are associated with weakening of the anti-bacterial and particularly anti-TB defense (Benoit et al., 2008). However, Mtb itself can inhibit M1 polarization to escape from host cell immunological killings, which suggest that the development of novel strategies that promoting M1 polarization of infected macrophages might be a promising method to enhance the innate defence of macrophages against intracellular Mtb.

**Figure 4:**
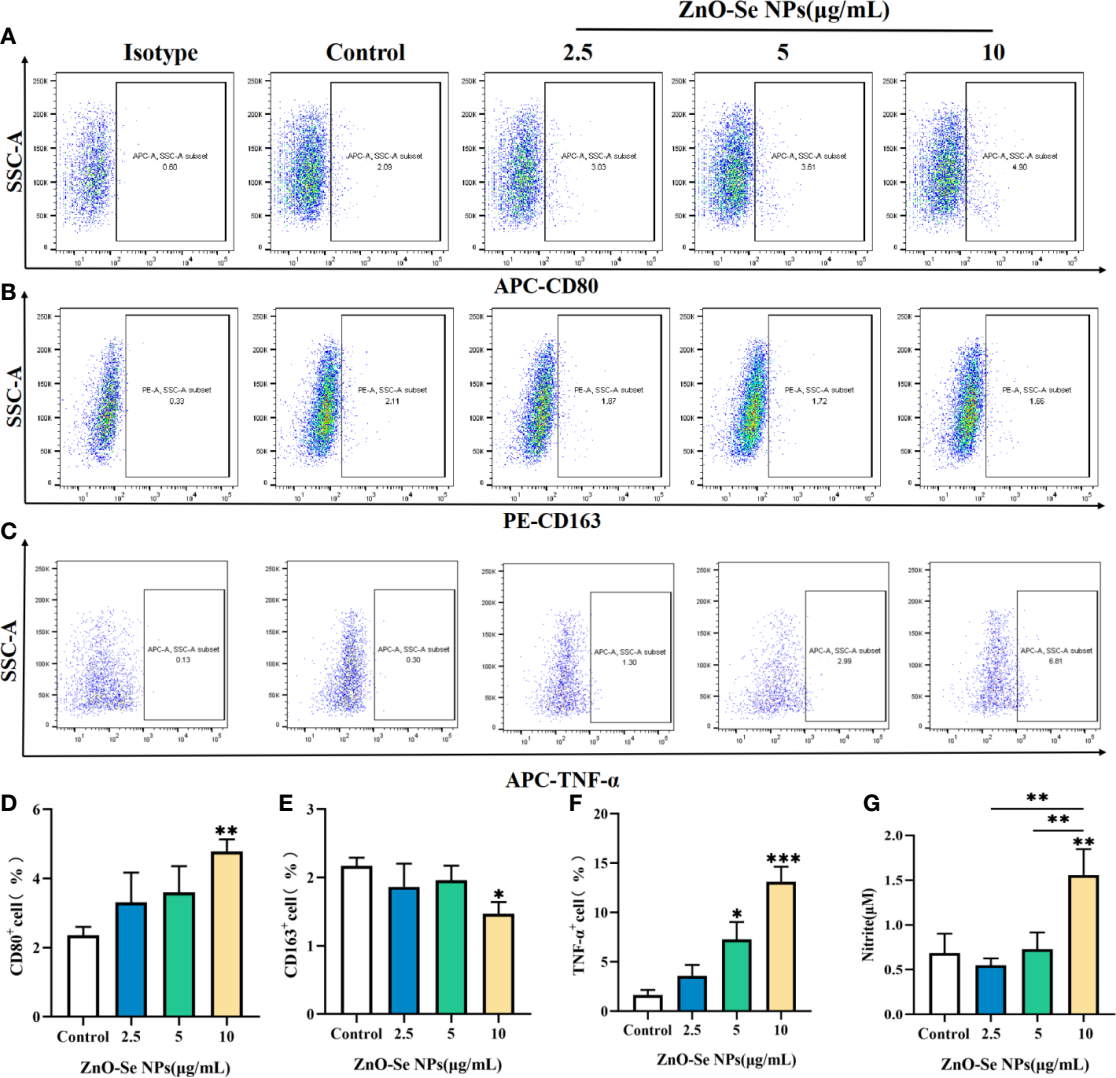
Effects of ZnO-Se NPs on the polarization of Mtb infected macrophages. **(A–C)** Typical flow cytometry analysis of **(A)** CD80 expression, **(B)** CD163 expression and **(C)** TNF-α expression in BCG infected THP-1 cells before and after ZnO-Se NPs treatment. **(D–F)** Statistical results of **(D)** CD80 expression, **(E)** CD163 expression and **(F)** TNF-α expression in BCG infected THP-1 cells before and after ZnO-Se NPs treatment. n=3, *p<0.05, **p<0.01,***p<0.001. **(G)** Effects of ZnO-Se NPs on the nitrite level in the supernatant of BCG infected THP-1 cells, n=3, **p<0.01.

To explore the potentials of ZnO-Se NPs to inhibit intracellular Mtb, we tested the effects of ZnO-Se NPs on polarization of Mtb infected macrophages. Firstly, we analyzed the expression of M1 macrophage marker CD80 and M2 macrophage marker CD163, which indicated that ZnO-Se NPs could increase the expression of CD80 (**Figures 4A, D**) and decrease the expression of CD163 (**Figures 4B, E**) in BCG infected THP-1 macrophages. Moreover, we also determined the expression of pro-inflammatory cytokines TNF-α, the typical cytokine produced by M1 macrophage, as well as the expression of anti-inflammatory IL-10, the typical cytokine produced by M2 macrophage (Mills, 2012; Viola et al., 2019). Although no significant changes were observed in IL-10 production (**Supplementary material Figure 3**), we found that ZnO-Se NPs treatment significantly increased TNF-α production in BCG infected THP-1 macrophages (**Figures 4C, F**), which further supported the conclusion that ZnO-Se NPs could promote M1 polarization in Mtb infected macrophages. As an important anti-TB cytokines, IFN-γ plays an important role in host defense against Mtb (Ghanavi et al., 2021). Therefore, we also tested the expression level of IFN-γ in BCG infected THP-1 macrophages, which demonstrated that ZnO-Se NPs treatment could also partially increase IFN-γ production in BCG infected THP-1 macrophages (**Supplementary material Figure 3**). As a critical anti-TB mediator generated from inducible NO synthase (iNOS) and an important anti-TB products of M1 macrophages, NO could further modulate macrophage functions to kill the intracellular Mtb (Jamaati et al., 2017). Thus, we also determined the nitrite concentration of BCG infected THP-1 macrophages, which demonstrated that ZnO-Se NPs treatment could dramatically increase the nitric oxide production of Mtb infected macrophages (**Figure 4G**). These results collectively suggested that ZnO-Se NPs could promote M1 polarization of Mtb infected macrophages with enhanced anti-TB cytokines production, which would benefit the intracellular Mtb inhibition by the host cell immunity.

### ZnO-Se NPs induce apoptosis and autophagy in Mtb infected macrophages by regulating intracellular ROS, mitochondria membrane potential and PI3K/Akt/mTOR signaling

**Figure 5:**
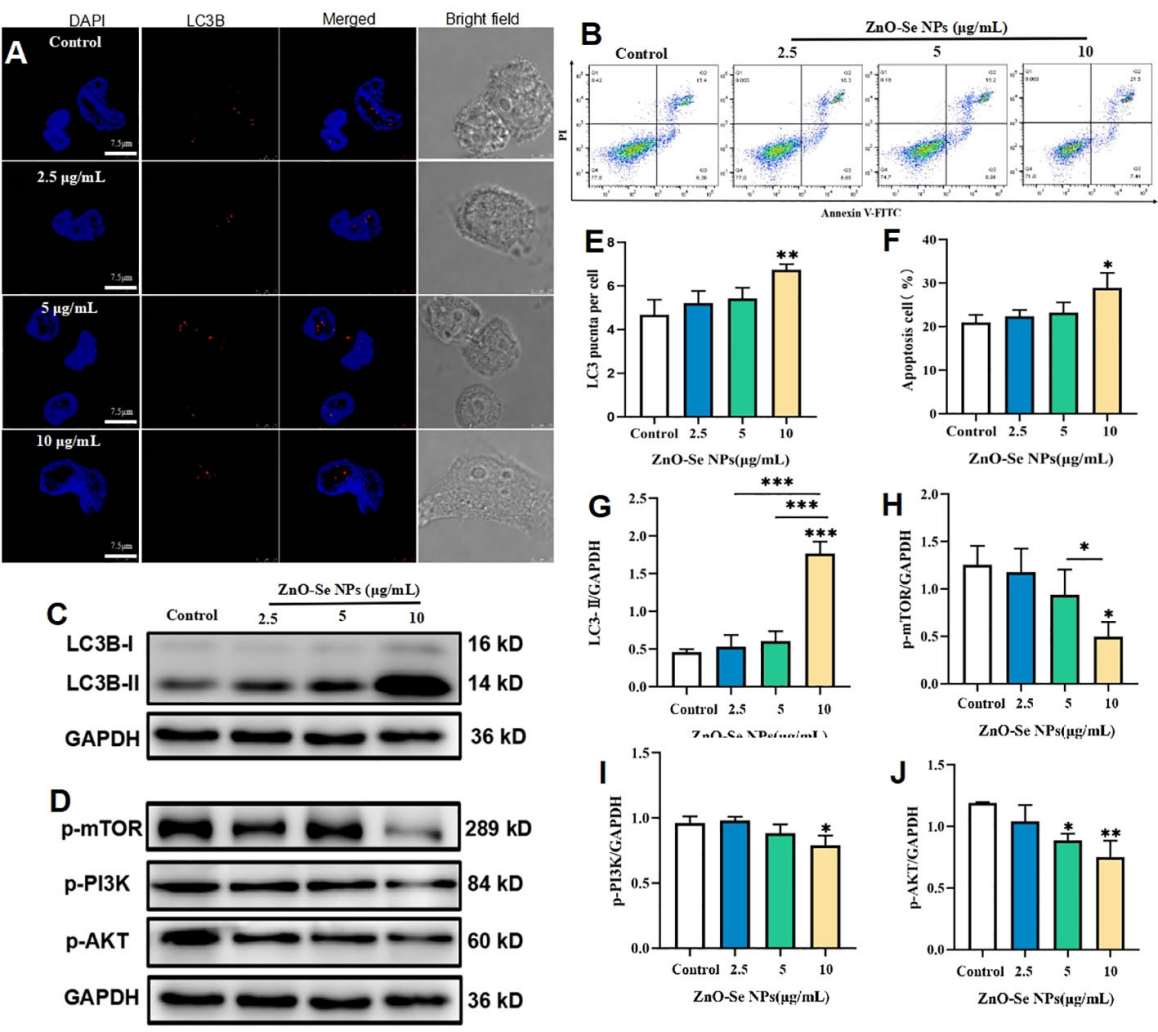
ZnO-Se NPs induce autophagy and apoptosis in Mtb infected macrophages by regulating PI3K/Akt/mTOR pathway. **(A)** Typical fluorescence imaging of LC3B staining in BCG infected THP-1 cells before and after ZnO-Se NPs treatment, scale bar: 7.5 μm. **(B)** Typical flow cytometry analysis of apoptosis for BCG infected THP-1 cells before and after ZnO-Se NPs treatment. **(C, D)** Typical western blot results for **(C)** LC3B expression and **(D)** the phosphorylation of mTOR, PI3K and AKT in BCG infected THP-1 cells before and after ZnO-Se NPs treatment. **(E)** Statistical results of LC3B punctas for BCG infected THP-1 cells before and after ZnO-Se NPs treatment. **(F)** Statistical results of apoptosis for BCG infected THP-1 cells before and after ZnO-Se NPs treatment. **(G)** Statistical results for the LC3B expression in BCG infected THP-1 cells before and after ZnO-Se NPs treatment. **(H)** Statistical results for the phosphorylation of mTOR in BCG infected THP-1 cells before and after ZnO-Se NPs treatment. **(I)** Statistical results for the phosphorylation of PI3K in BCG infected THP-1 cells before and after ZnO-Se NPs treatment. **(J)** Statistical results for the phosphorylation of AKT in BCG infected THP-1 cells before and after ZnO-Se NPs treatment, n≥3, *p<0.05, **p<0.01, ***p<0.001.

For macrophages, apoptosis and autophagy have been recognized as an important innate defense mechanisms against intracellular Mtb, (Xu et al., 2014; Lam et al., 2017). To escape from the killing effects of host cell immunity, virulent Mtb can produce some bacterial products to inhibit apoptosis and autophagy of macrophages (Xu et al., 2014; Lam et al., 2017). Cell autophagy, a self-degradative process that important to remove the unnecessary or dysfunctional components through a lysosome-dependent regulated machanism, has been widely proved to function as an innate immunological responses for removing the intracellular pathogens (Xu et al., 2014; Lam et al., 2017). Our and other’s groups have previously introduced the strategy of promoting autophagy to kill and inhibit intracellular Mtb (Franco et al., 2017; Luo et al., 2021; Yang et al., 2021), all demonstrating the promising potentials of autophagy regulations for anti-TB treatments. As shown in **Figures 5A, E**, 10 μg/mL of ZnO-Se NPs treatment induced the formation of LC3B punctas in BCG infected THP-1 macrophages. Moreover, 10 μg/mL of ZnO-Se NPs were also found to increase the expression of LC3B-II in BCG infected THP-1 macrophages (**Figures 5C, G**), which collectively demonstrated that ZnO-Se NPs could promote the autophagy of Mtb infected macrophages. However, same dosages of ZnO-Se NPs didn’t induce significant increase of LC3B-II in normal THP-1 macrophages without BCG infection (**Supplementary Material Figure 4**). These results indicated the selective autophagy induction effects of ZnO-Se NPs in Mtb infected macrophages, but the exact mechanisms for this selectivity remained to be further investigated in the following work.

As a kind of programmed cell death, apoptosis in pathogen infected host cells is a deadly cell fate that the host cells scarify themselves to kill the intracellular Mtb, which could effectively avoid the replication and escaping of intracellular Mtb to further infect other cells (Xu et al., 2014; Lam et al., 2017). Thus, using some active agents to induce Mtb infected cell apoptosis might be served as a promising strategy to killing the intracellular Mtb (Mahajan et al., 2012). As shown by the flow cytometry analysis, ZnO-Se NPs could significantly increase the apoptosis of BCG infected THP-1 macrophages (**Figures 5B, F**), however, same dosages of ZnO-Se NPs didn’t induce significant apoptosis in normal THP-1 macrophages without BCG infection (**Supplementary Material Figure 4**). Although the detailed mechanisms remain to be further investigated, these results suggested that ZnO-Se NPs could selectively induce apoptosis in Mtb infected macrophages, which suggested that apoptosis played a critical role in ZnO-Se NPs inhibited intracellular Mtb in macrophages.

PI3K/Akt/mTOR is a major intracellular signaling pathway that is crucial to many aspects of cell growth/survival, and also play important roles in physiological as well as in pathological condition (Porta et al., 2014; Mohseni et al., 2021). Due to the critical roles of PI3K/Akt/mTOR signaling events in cell proliferation, apoptosis and autophagy, we further determined the effects of ZnO-Se NPs treatment on the phosphorylation of AKT, mTOR and PI3K in BCG infected THP-1 macrophages. As shown in **Figures 5D, H–J**, the phosphorylation of AKT, mTOR and PI3K in BCG infected THP-1 macrophages were significantly inhibited after 10 μg/mL of ZnO-Se NPs treatment. It has been widely proved that the inhibition of PI3K/Akt/mTOR pathway would promote cell autophagy and apoptosis (Kang et al., 2012; Heras-Sandoval et al., 2014). Therefore, our results indicated that ZnO-Se NPs induced apoptosis and autophagy of BCG infected THP-1 macrophages were closely associated with the inhibition effects on ZnO-Se NPs on the PI3K/Akt/mTOR signaling pathway.

**Figure 6:**
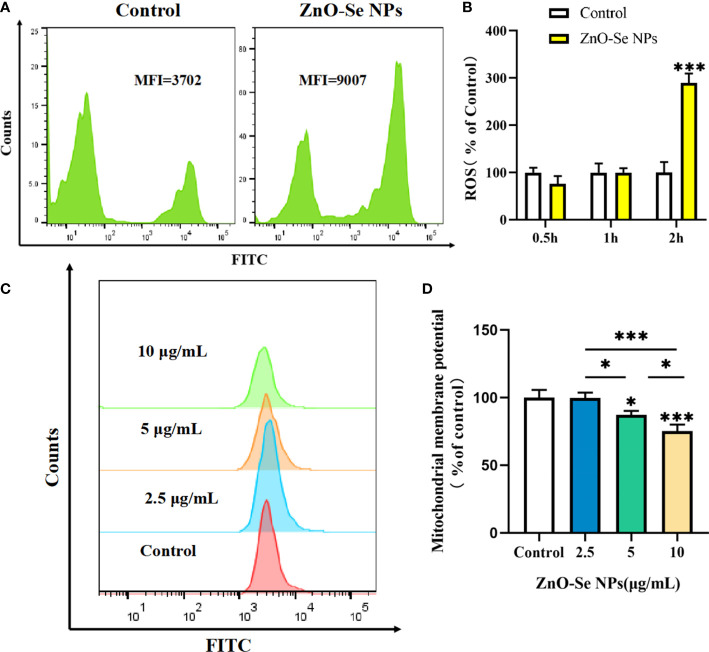
ZnO-Se NPs induce autophagy and apoptosis in Mtb infected macrophages by increasing intracellular ROS level and disrupting mitochondrial membrane potential. **(A)** Typical flow cytometry analysis of ROS level for BCG infected THP-1 cells before and after 2.5 μg/mL ZnO-Se NPs treatment. **(B)** Statistical results of ROS level for BCG infected THP-1 cells before and after ZnO-Se NPs treatment, n=3, ***p<0.001. **(C)** Typical flow cytometry analysis of mitochondria membrane potential for BCG infected THP-1 cells before and after ZnO-Se NPs treatment. **(D)** Statistical results of mitochondria membrane potential for BCG infected THP-1 cells before and after ZnO-Se NPs treatment, n=3, *p<0.05, ***p<0.001.

Cell apoptosis and autophagy are also regulated by some important by-products of cells, such as the intracellular ROS. ROS, a number of reactive molecules and free radicals derived from molecular oxygen, has been widely reported to play important roles in intracellular pathogen killings, including Mtb (Dharmaraja, 2017; Shastri et al., 2018). By analyzing the intracellular ROS, we found that ZnO-Se NPs treatment could rapidly increase the intracellular ROS level in BCG infected macrophages in 2 h (**Figures 6A, B**). Mitochondria are an important source of ROS, while the excessive ROS production contributes to mitochondrial damage in a range of pathologies and is also important in redox signalling from the organelle to the rest of the cell (Ott et al., 2007). Here, we also found that ZnO-Se NPs could significantly decrease the mitochondria membrane potential of BCG infected macrophages (**Figures 6C, D**). However, the results obtained on normal THP-1 cells indicated that same dosages of ZnO-Se NPs treatment didn’t induce significant decreases of mitochondria membrane potential in normal THP-1 macrophages without BCG infection (**Supplementary Material Figure 4**). We have previously proved that Se NPs could promote the apoptosis and autophagy of Mtb infected macrophages by the regulations of intracellular ROS and mitochondria membrane potential (Pi et al., 2020). Taking all the above results into account, we found that ZnO-Se NPs could promote the apoptosis and autophagy of Mtb infected macrophages by regulating PI3K/Akt/mTOR signaling, increasing the intracellular level and disrupting mitochondria structure and functions.

### ZnO-Se NPs significantly inhibit intracellular Mtb growth in Mtb infected macrophages

**Figure 7:**
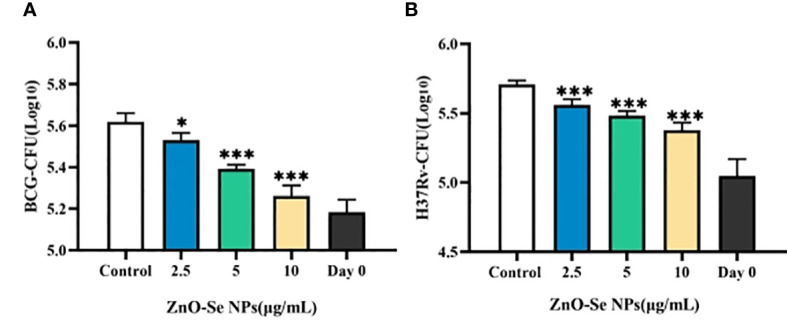
Inhibition effects of ZnO-Se NPs on the intracellular Mtb. **(A)** Effects of ZnO-Se NPs on BCG growth in BCG infected THP-1 cells, *p<0.05, ***p<0.001. **(B)** Effects of ZnO-Se NPs on H37Rv growth in H37Rv infected THP-1 cells, *p<0.05, ***p<0.001.

To finally validate the anti-TB effects of ZnO-Se NPs for potential anti-TB treatment, we further determined the effects of ZnO-Se NPs on the growth of intracellular Mtb. As shown in **Figure 7A**, ZnO-Se NPs could significantly inhibit BCG growth in BCG infected THP-1 macrophages. Moreover, we also tested their effects on the virulent Mtb strain H37Rv, which also indicated the strong inhibition effects of ZnO-Se NPs against H37Rv in H37Rv infected THP-1 macrophages (**Figure 7B**). These results collectively suggested that ZnO-Se NPs could indeed act as a kind of novel anti-TB agents against intracellular Mtb. We also compared the intracellular Mtb inhibition effects of ZnO-Se NPs with the widely used clinical anti-TB drug rifampicin. The high dosage (10 μg/mL) of ZnO-Se NPs treatment resulted in similar inhibition effects of low dosage (50 ng/mL) of rifampicin treatment (**Supplementary material Table 1**). Although much higher dosage of ZnO-Se NPs is needed for intracellular Mtb inhibition compared with rifampicin, our results have indicated the potential of ZnO-Se NPs for anti-TB treatments.

Mtb is a typical intracellular pathogenic bacteria that very difficult to be killed or cleared with high efficiency. Although ZnO-Se NPs have showed direct killing effects against Mtb (As shown in **Figures 2**, **3**), these direct killing effects would be dramatically limited for intracellular Mtb killings. The intracellular Mtb are hidden in the intracellular components, which could avoid the direct interaction between ZnO-Se NPs and Mtb to restrict the intracellular anti-TB effects of ZnO-Se NPs. Thus, we think the host cell immunological tuning ability of ZnO-Se NPs to stimulate anti-Mtb innate immune responses would be the primary mechanism of intracellular protection against Mtb in infected THP1 cells.

It’s a pity that we didn’t use primary macrophages, such as BMDMs, to establish Mtb infected primary macrophgae model, which would be helpful to better interpret the ability of ZnO-Se NPs to inhibit the intracellular Mtb growth and intracorporal Mtb growth. Moreover, the anti-TB effects of ZnO-Se NPs also need to be further confirmed in Mtb infected animal model, which would not only be helpful to test the ability of ZnO-Se NPs to clear intracorporal Mtb, but would also be conducive to explore their ability to reduce the pathological damage induced by ZnO-Se NPs. We will further confirm these effects of ZnO-Se NPs in our future works.

## Conclusion

In this work, we introduced a kind of novel anti-TB agents, naming ZnO-Se NPs, which was prepared by the hybridization of zinc oxide and selenium nanoparticles. The obtained ZnO-Se NPs showed spherical core-shell morphology and strong stability with average diameters of 90 nm. Interestingly, ZnO-Se NPs showed strong killing effects against extracellular Mtb, including BCG and the virulent H37Rv, by disrupting the ATP production, increasing the intracellular ROS level and destroying the membrane structures. More importantly, ZnO-Se NPs could also inhibit intracellular Mtb growth by promoting M1 polarization to increase the production of antiseptic nitric oxide in Mtb infected macrophages. ZnO-Se NPs inhibited intracellular Mtb was also closely associated with their ability to induce apoptosis and autophagy of Mtb infected macropahegs by increasing the intracellular ROS, disrupting mitochondria membrane potential and inhibiting PI3K/Akt/mTOR signaling. The proposed ZnO-Se NPs with synergetic anti-TB effects by combining their Mtb killing effects and host cell immunological inhibition effects were expected to serve as novel anti-TB agents for the development of anti-TB strategy.

## Data availability statement

The raw data supporting the conclusions of this article will be made available by the authors, without undue reservation.

## Author contributions

WL, SF and KL performed the experiments, analyzed the data and drafted the manuscript. YH, YC, JZ, HJ, YZ, YR, HL, FY, CW, DZ, ZF helped to design/preform the experiments or data analysis. BZ, J-FX and JP were responsible for leading this work, designing this study and revising the manuscript. All authors contributed to the article and approved the submitted version.
