# Supplementary material for: Engineering zinc oxide hybrid selenium nanoparticles for synergetic anti-tuberculosis treatment by combining Mycobacterium tuberculosis killings and host cell immunological inhibition
Source: Front Cell Infect Microbiol. 2023 Jan 26;12:1074533. doi: 10.3389/fcimb.2022.1074533 (PMC9908760; doi:10.3389/fcimb.2022.1074533)
Supplement: Supplementary file 1 [file DataSheet_1.pdf]

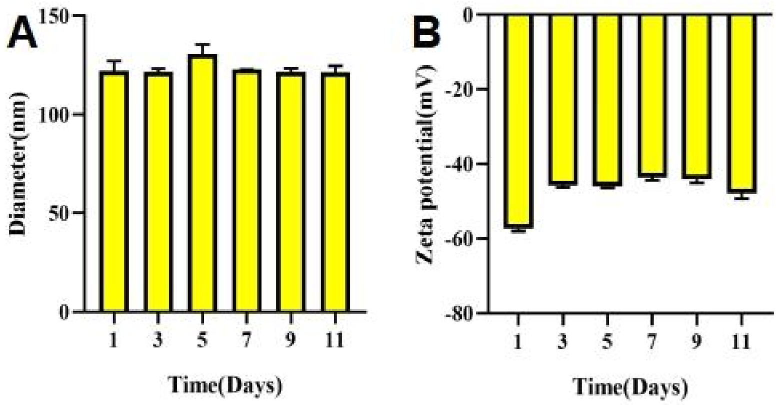

Supplementary materials. Fig.1 Stability of ZnO-Se NPs determined by dynamic light scattering (DLS). (A) Size changes of ZnO-Se NPs by DLS in 10 days storage. (B) Zeta potential changes of ZnO-Se NPs by DLS in 10 days storage.

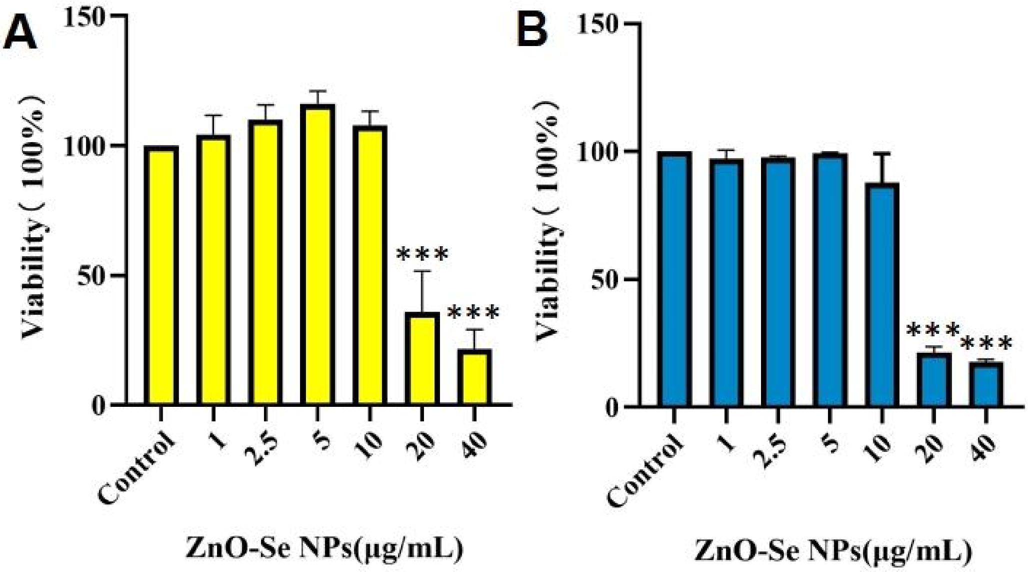

Supplementary materials. Fig.2 Effects of ZnO-Se NPs treatment on the viability of (A) THP-1 cells and (B) Raw264.7 cells, n=3, *p<0.05, ***p<0.001. THP-1 cells were seeded into 96 well plates in a density of 10000 cell per well with the stimulation of 100 nM PMA for 24 h. Cells were then incubated with different concentration of ZnO-Se NPs for 24 h. Raw264.7 cells were seeded into 96 well plates in a density of 10000 cell per well for 24 h. After ZnO-Se NPs treatment, CCK-8 solution were added into each well for 4 h incubation. Then, the Microplate reader (Tecan, Switzerland) was used to measure absorbance at 450 nm.

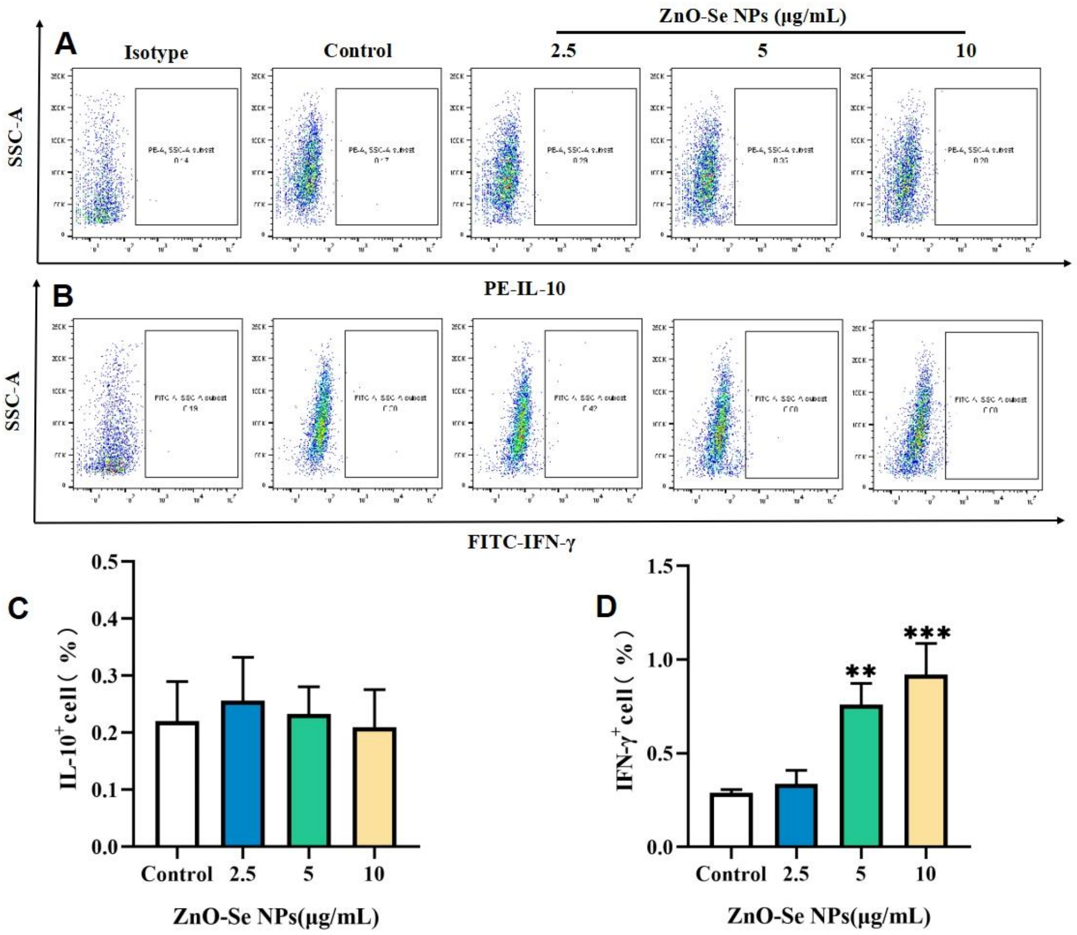

Supplementary materials. Fig.3 Effects of ZnO-Se NPs on the IL-10 and IFN-γ production of Mtb infected macrophages. (A-B) Typical flow cytometry analysis of (A) IL-10 level and (B) IFN-γ level in BCG infected THP-1 cells before and after ZnO-Se NPs treatment. (C-D) Statistical results of (C) IL-10 level and (D) IFN-γ level in BCG infected THP-1 cells before and after ZnO-Se NPs treatment, n=3, $^{**}p<0.01$,$^{***}p<0.001$.

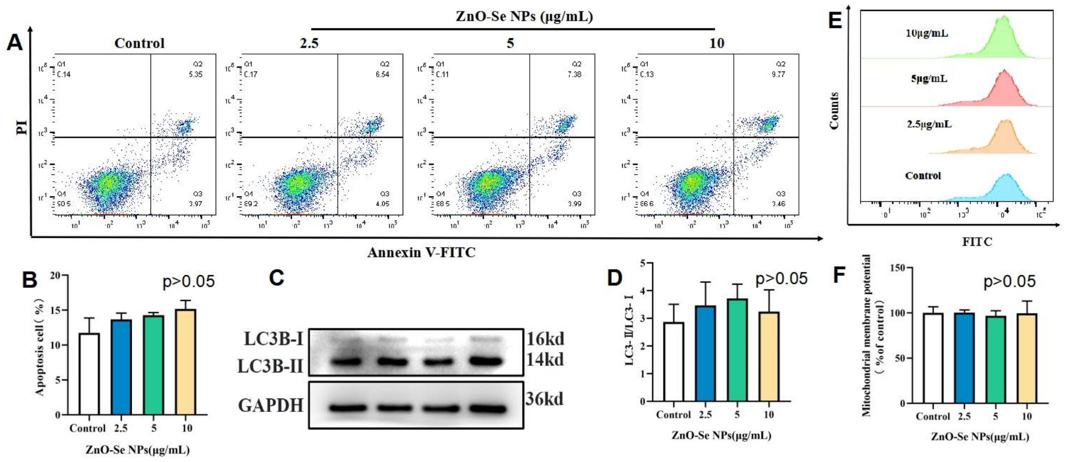

Supplementary materials. Fig.4 Effects of ZnO-Se NPs on the apoptosis, autophagy, and mitochondiral membrane potential of normal THP-1 macrophages without Mtb infection. (A) Typical flow cytometry analysis of apaoptosis in normal THP-1 cells before and after ZnO-Se NPs treatment. (B) Statistical results of apaoptosis in normal THP-1 cells before and after ZnO-Se NPs treatment. (C) Typical western blot results for LC3B expression in normal THP-1 cells before and after ZnO-Se NPs treatment. (D) Statistical results for LC3B expression in normal THP-1 cells before and after ZnO-Se NPs treatment. (E) Typical flow cytometry analysis of mitochondria membrane potential for normal THP-1 cells before and after ZnO-Se NPs treatment. (F) Statistical results of mitochondria membrane potential for normal THP-1 cells before and after ZnO-Se NPs treatment.

Supplementary materials. Table 1. Inhibition effects of ZnO-Se NPs, rifampicin on the intracellular BCG and H37Rv in infected macrophages. THP-1 cells were infected with BCG or H37Rv with a MOI of 1 for 24 h or 4 h, respectively. The infected cells were then treated with ZnO-Se NPs or rifampicin for 72 h. Cell lysis were plated on the 7H11 plates for 3-4 weeks incubation and then used for CFU counting. The results for the inhibition rates were calculated as the (CFU counts for drug treated group/CFU counts for control group without drug treatment)*100%±S.D..

Treatment	Inhibition rates on intracellular BCG in THP-1 cells	Inhibition rates on intracellular H37Rv in THP-1 cells
ZnO-Se NPs (10 μg/mL)	44.0±5.0%	49.4±3.0%
Rifampicin (50 ng/mL)	51.1±4.0%	54.1±8.1%
